# The Composition of Hyperacute Serum and Platelet-Rich Plasma Is Markedly Different despite the Similar Production Method

**DOI:** 10.3390/ijms20030721

**Published:** 2019-02-08

**Authors:** Dorottya Kardos, Melinda Simon, Gabriella Vácz, Adél Hinsenkamp, Tünde Holczer, Domonkos Cseh, Adrienn Sárközi, Kálmán Szenthe, Ferenc Bánáti, Susan Szathmary, Stefan Nehrer, Olga Kuten, Mariana Masteling, Zsombor Lacza, István Hornyák

**Affiliations:** 1 Institute of Clinical Experimental Research, Semmelweis University, Budapest 1094, Hungary; melinda.simon@orthosera.com (M.S.); vaczgabi@gmail.com (G.V.); adel.hinsenkamp@orthosera.com (A.H.); mastelin@umich.edu (M.M.); zsombor.lacza@orthosera.com (Z.L.); istvan.hornyak@orthosera.com (I.H.); 2 Department of Laboratory Medicine, Semmelweis University, Budapest 1089, Hungary; holczer.tunde@med.semmelweis-univ.hu; 3 Department of Physiology, Semmelweis University, Budapest, 1094 Hungary; cseh.domonkos@med.semmelweis-univ.hu (D.C.); sarkoziadrienn88@gmail.com (A.S.); 4 RT-Europe Non-profit Research Center, Mosonmagyaróvár 9200, Hungary; kszenthe@rt-europe.org (K.S.); fbanati@rt-europe.org (F.B.); 5 Galenbio Ltd., Mosonmagyaróvár 9200, Hungary; sszathmary@galenbio.com; 6 Danube University, Center for Regenerative Medicine, Krems an der Donau 3500, Austria; stefan.nehrer@donau-uni.ac.at (S.N.); olga.kuten@orthosera.com (O.K.); 7 Faculdade de Engenharia da Universidade do Porto, Universidade do Porto, Porto 4200-465, Portugal; 8 University of Physical Education, Institution of Sport and Health Sciences, Budapest 1123, Hungary; 9 Orthosera GmbH, Krems an der Donau 3500, Austria

**Keywords:** hyperacute serum, platelet-rich plasma, blood derived products, composition

## Abstract

Autologous blood derived products, such as platelet-rich plasma (PRP) and platelet-rich fibrin (PRF) are widely applied in regenerative therapies, in contrast to the drawbacks in their application, mainly deriving from the preparation methods used. Eliminating the disadvantages of both PRP and PRF, hyperacute serum (HAS) opens a new path in autologous serum therapy showing similar or even improved regenerative potential at the same time. Despite the frequent experimental and clinical use of PRP and HAS, their protein composition has not been examined thoroughly yet. Thus, we investigated and compared the composition of HAS, serum, PRP and plasma products using citrate and EDTA by simple laboratory tests, and we compared the composition of HAS, serum, EDTA PRP and plasma by Proteome Profiler and ELISA assays. According to our results the natural ionic balance was upset in both EDTA and citrate PRP as well as in plasma. EDTA PRP contained significantly higher level of growth factors and cytokines, especially platelet derived angiogenic and inflammatory proteins, that can be explained by the significantly higher number of platelets in EDTA PRP. The composition analysis of blood derivatives revealed that although the preparation method of PRP and HAS were similar, the ionic and protein composition of HAS could be advantageous for cell function.

## 1. Introduction

Autologous blood derived products, particularly platelet concentrates, are widely applied nowadays in different regenerative therapies which include wound healing, orthopedics, and dentistry [1,2,3,4,5]. Platelet derived growth factors are able to enhance soft and hard tissue regeneration by increasing cell migration and proliferation, and decreasing the rate of inflammation [6,7]. The most commonly used platelet concentrate products are PRP (platelet-rich plasma)—also known as first generation blood product—and PRF (platelet-rich fibrin), which is known as second generation blood product. PRP has a wide range of application in wound healing, cartilage, bone, musculoskeletal regeneration, oral surgery, dentistry, and cosmetics [8,9,10,11,12,13]. Moreover, PRP has been successfully applied in treating endometriosis, chronic skin ulcer and vitiligo in earlier studies [14,15]. Nevertheless, there are some drawbacks of using PRP [16]. For example, the lack of uniformity in PRP preparation methods [4,17] which is mainly caused by the treatment of blood with different anticoagulants like EDTA and citrate, which are the most preferred ones in PRP preparation. The information in the literature on the optimal anticoagulant for PRP preparation is contradictory. On the basis of previous studies, both EDTA and citrate (sodium citrate, acid citrate dextrose) have a negative effect on the balance of ionic content in the plasma fraction because of their chelating mechanism [18]. Higher platelet numbers can be obtained by using EDTA compared to citrate [19,20], however the mean platelet volume (MPV)—which is a marker of platelet function—can be higher than citrate using EDTA [20]. Other studies have claimed that platelet aggregation was inhibited more efficiently by using EDTA than acid citrate dextrose solution [19]. Using EDTA, spontaneous platelet activation can be avoided because EDTA has a stronger complex capacity with divalent cations than citrate. For the activation of citrate and EDTA, PRP thrombin and calcium–chloride or temperature activation can be used. [21] The addition of bovine thrombin during PRP preparation may lead to cross-reaction with human factor Va causing bleeding disorder, but it is more effective than activating PRP by repeated freezing and thawing [16].

With PRF the major disadvantages of PRP can be avoided. In addition, growth factors and cytokines are released from platelets over a longer period of time, whereas in PRP these factors are released immediately into the site of application [22,23]. Nevertheless, PRF is not capable of replacing PRP in all therapeutic areas because of its compact three-dimensional structure, which hinders its application as an injection [24,25,26]. Consequently, PRF is mainly used for replacing injured tissues in oral and dental surgery, orthopedics, and wound healing [27,28,29,30,31].

SPRF (serum from platelet-rich fibrin) or HAS (hyperacute serum) was developed to avoid the limitations of both PRF and PRP. The preparation method is the same as in the case of PRF, but the serum is squeezed out from the PRF clot at the end of the procedure [32]. Thus, it does not contain any anticoagulant or thrombin, and the final product is liquid. Based on our previous results, HAS has a better cell proliferative effect on mesenchymal stem cells, osteoblasts, and osteoarthritic chondrocytes compared to EDTA PRP [33]. Furthermore, HAS promotes MSCs lineage shift towards the osteoblastic line and results in a better preserved bone marrow structure in bone marrow explants compared to EDTA PRP treatments [32,34].

Despite the positive results of HAS in cell proliferation and migration, the composition of HAS has not been reported so far. However, angiogenic proteins were measured earlier both in HAS and PRP by Proteome Profiler, showing that EDTA PRP has more angiopoietic components, whereas HAS has more anti-angiopoietic components [35]. In the present study we investigated and compared the composition of HAS, PRP, blood serum, and plasma to understand the differences in the cell proliferative and regenerative effect of HAS and PRP. In the laboratory tests EDTA and citrate anticoagulated PRP were also compared. However, due to the more homogenous blood fraction separation that was achieved using EDTA, and based on the earlier cell culture experiments, the semi-quantitative and quantitative analysis was only done with PRP that was anticoagulated with EDTA in order to allow consequent results and interpretation.

## 2. Results

First, we measured the concentration of relevant metal ions, inorganic phosphate, the activity of ALP enzyme, and the quantity of cellular blood components in the serum and plasma samples by a general laboratory test. EDTA and citrate PRP were activated by thrombin and CaCl_2_ with heparin together to prevent coagulation, thus the chelation capacity of both K_3_EDTA and sodium citrate was reduced in PRP.

**Figure 1:**
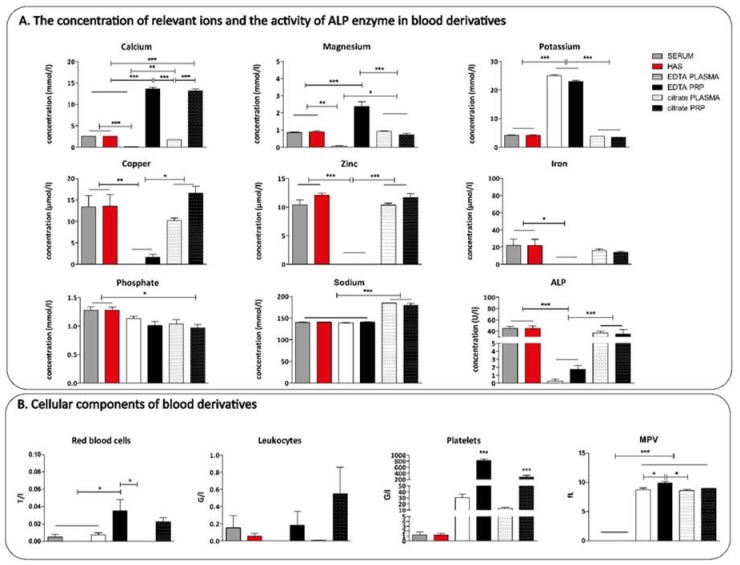
Quantitative determination of the concentration of relevant ions and the activity of alkaline phosphatase (ALP) enzyme (**A**) and the number of red blood cells, leukocytes and platelets in the serum and plasma fractions (**B**), *n* = 4. The significance level was *p* > 0.05, where * means that *p* is between 0.01 and 0.05, ** means that *p* is between 0.01 and 0.001, and *** means that *p* is lower than 0.001.

The concentration of calcium ions was significantly higher in EDTA and citrate PRP, and lower in EDTA and citrate plasma compared to the serum fractions. Both anticoagulants effectively chelate calcium and citrate is more effective in forming complexes with magnesium ions. The level of potassium ions was higher in EDTA plasma fractions due to the potassium content of K_3_EDTA. The concentration of copper, zinc, and iron ions was also significantly lower in EDTA plasma and EDTA PRP due to the chelating ability of K_3_EDTA. Sodium ion concentration was increased due to the sodium content of citrate while phosphate content was only significantly influenced using citrate PRP. The activity of ALP enzymes was also reduced significantly because EDTA naturally chelates Mg^2+^ and Zn^2+^ which have a crucial role in the structural stability of ALP enzyme (Figure 1A).

Sodium citrate has a weaker complex capacity with divalent cations than EDTA, thus the ion content of citrate plasma fractions was not influenced significantly except calcium ions, which were added both to citrate and EDTA PRP for activation. Cellular blood components were measured before activating PRP because activated platelets might have been disrupted. The number of leukocytes was the highest in citrate PRP and the standard error of mean was high in the case of EDTA and citrate PRP as well. The number of platelets was higher in EDTA PRP compared to citrate PRP. The platelet number was extremely low in EDTA and citrate plasma, serum and HAS. Mean platelet volume (MPV), which is a marker of platelet function, was similar in EDTA and citrate PRP. (Figure 1B). For further examination EDTA plasma and PRP were used because a higher number of platelets could be isolated by EDTA than citrate with similar MPV values. Furthermore, EDTA enabled the most homogenous separation and therefore EDTA was used as an anticoagulant in our previous studies where the cell proliferation rate of different cell types was investigated.

**Figure 2:**
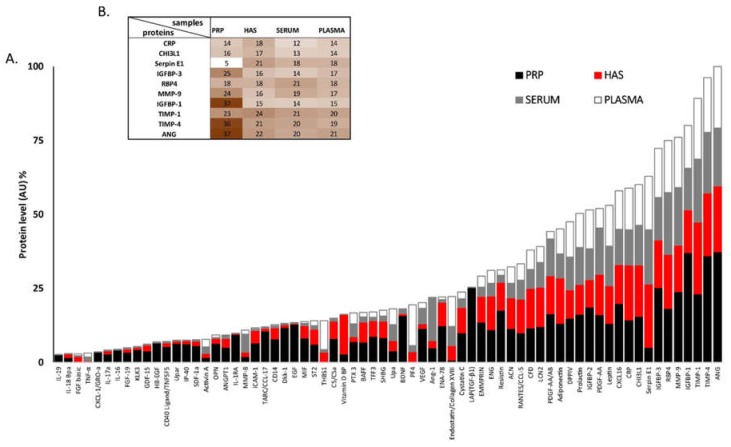
Semi-quantitative Proteome Profiler analysis of serum, hyperacute serum (HAS), plasma, and platelet-rich fibrin (PRP). On the bar chart proteins exceeding 2% (AU) of the total protein content (**A**) are presented. The level of the top 10 angiogenic proteins and cytokines are presented on a heat map. (**B**) The level of the proteins is expressed in % compared to the combined arbitrary unit of ANG that was considered to be 100%. *n* = 8.

For mapping the composition of serum, HAS, EDTA plasma, and PRP (Figure 2A), 138 different known cytokines and growth factors were screened using antibody-based dot-blot assays. The results were presented as relative values compared to the positive control for the secondary antibody. From the 138 cytokines and growth factors selected as known paracrine mediators of tissue regeneration, vascularization or inflammation, 82 proteins were neglected (AU < 2%) thus 56 proteins (AU > 2%) are presented in Figure 2A [35]. The whole data set containing the 138 cytokines and growth factors is shown in Table S1. We observed a general trend that the overall concentration of active molecules was the highest in PRP, followed by HAS, plasma, and serum, respectively (see in Figure 2A and Table S1). There were clear differences in the proteome profiler patterns of the blood derivatives, although the proteome profiler is not reliable enough for quantitative measurements and for the statistic comparison of the blood derivates. Thus, on the basis of these results and the literature [6,36,37,38,39] we set out to quantify the key inflammation related cytokines and proteins with ELISA or Luminex assays.

**Figure 3:**
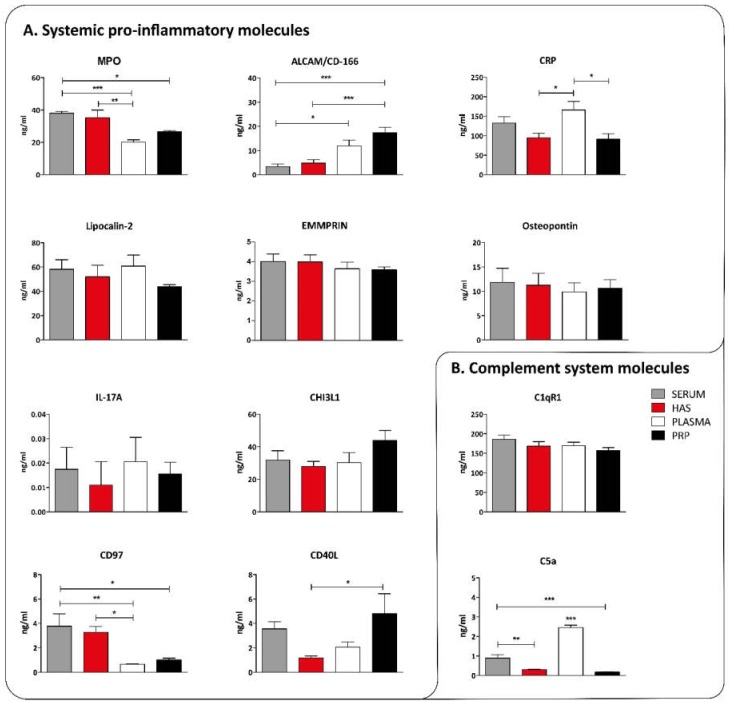
Concentration of systemic pro-inflammatory molecules (**A**) and complement system related molecules (**B**) in blood derivatives, *n* = 8. The significance level was *p* > 0.05, where * means that *p* is between 0.01 and 0.05, ** means that *p* is between 0.01 and 0.001, and *** means that *p* is lower than 0.001.

For quantitative protein analysis, Luminex and ELISA assays were used where systemic pro-inflammatory molecules, complement system molecules, platelet-derived inflammation, angiogenesis related molecules, and anti-inflammatory molecules were investigated. Lipocalin-2, EMMPRIN (CD147), Osteopontin, IL-17A, and Chitinase-3-like protein 1 (CHI3L1) are similarly present in all blood derivates. While CD97 and Myeloperoxidase (MPO) were higher in serum derivates, ALCAM was higher in plasma derivates. The concentration of CD40L was elevated in PRP compared to HAS, and CRP was significantly higher in plasma than in HAS and PRP (Figure 3A). From the complement system we measured C5a that showed the highest levels in plasma and C1qR1 which was highly elevated in all cases (Figure 3B).

**Figure 4:**
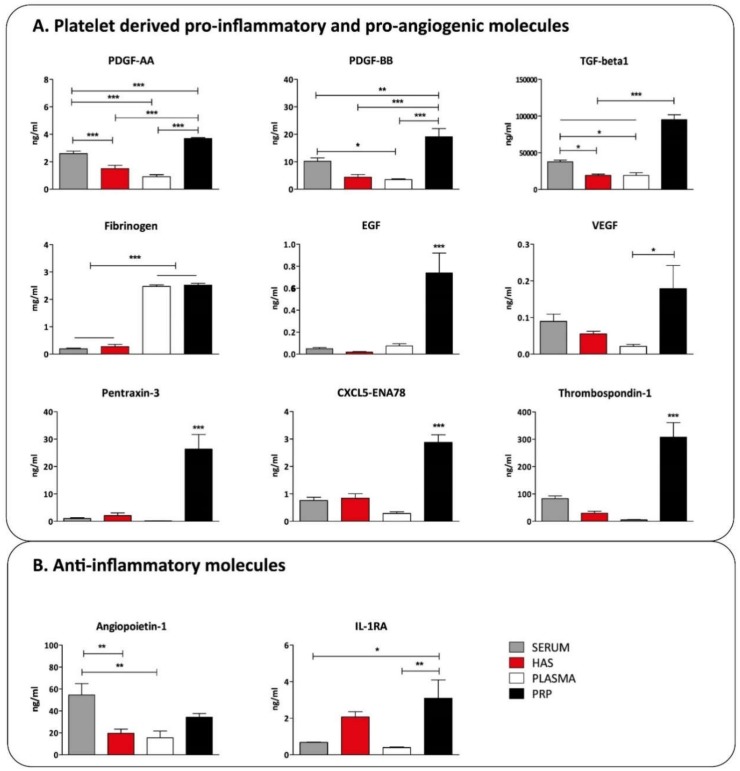
The concentration of platelet-derived inflammatory molecules (**A**) and anti-inflammatory molecules (**B**) in blood derivatives, *n* = 8. The significance level was *p* > 0.05, where * means that *p* is between 0.01 and 0.05, ** means that *p* is between 0.01 and 0.001, and *** means that *p* is lower than 0.001.

Platelet-derived pro-inflammatory cytokines and growth factors showed a very clear pattern: they were higher in PRP compared to other blood derivatives. In contrast, the fibrin/fibrinogen level was high both in plasma and PRP compared to serum and HAS, as the latter two were already coagulated and the clot was removed (Figure 4A). The anti-inflammatory cytokine IL-1RA was elevated in PRP and HAS. Angiopoietin-1 showed the highest level in serum but was present in all other blood derivatives in effective concentrations (Figure 4B).

## 3. Discussion

In the present study we investigated and compared the composition of different blood derivatives, especially EDTA, citrate PRP and HAS. On the basis of the literature PRP has excellent regenerative effects in numerous clinical applications, such as bone and cartilage regeneration, in osteoarthritis, in dental and oral surgery, or in musculoskeletal regeneration [8,9,11,40]. However, there are numerous drawbacks in the application of PRP mainly caused by certain steps of the preparation methods which are not present in the preparation of HAS [19,20].

First, the blood derivatives were analyzed by laboratory tests, where the number of cellular blood components, the concentration of relevant ion contents and the activity of ALP enzymes were measured. The concentration of ions, such as iron, copper, and zinc which are all influenced by the chelating mechanism of EDTA, were significantly lower, whereas potassium was higher in PRP and plasma because K_3_EDTA was used as an anticoagulant. The upset of the natural ionic balance due to EDTA PRP treatment may result in unexpected side effects when using it inside injured and/or inflamed tissue. As EDTA chelated both Zn^2+^ and Mg^2+^ the activity of ALP enzymes was reduced significantly. ALP has an important role in bone healing and regeneration, thus the reduced enzymatic activity has a negative effect in the case of injecting PRP into an osteoarthritic or injured joint [41]. The ionic balance of citrate PRP was much better, except for calcium ions which were added to PRP for platelet activation, and sodium because of the addition of sodium citrate. However, a significantly lower number of platelets could be isolated using citrate than EDTA, and the number of leukocytes was also much higher in citrate PRP.

The standard error of mean was high, indicating that the level of red blood cells and leukocytes in the samples depend mainly on the preparation method. During the preparation of HAS, the red blood cell containing fraction was cut away from the bottom of the PRF clot, while in case of PRP preparation, plasma fraction was removed by pipetting it carefully from the top of the red blood cell containing fraction. Using this isolation procedure, the PRP fraction contains some red blood cell fraction. The plasma isolation procedure is more complicated when citrate is used as an anticoagulant because the plasma and red blood cell fraction overlaps after centrifugation and the boundary could not be defined in a uniform manner. The mean platelet volume was similar in EDTA and citrate PRP, which indicates that platelet function was not influenced significantly by EDTA in our experiments. Using citrate, much better ionic composition can be achieved while the composition of blood shaped elements is much less favorable than EDTA PRP. On the basis of these results, and because only EDTA PRP was used in our previous studies for proteome profiler and luminex analysis, only EDTA PRP and plasma were used.

The level of angiogenic proteins [35] and cytokines was screened by semi-quantitative proteome profiler analysis. This method is not accurate enough for statistic comparison but was sufficient to determine that the overall concentration of proteins was the highest in EDTA PRP, followed by HAS, plasma, and the lowest was in serum. For quantitative protein measurement, inflammation-related molecules were chosen based on the proteome profiler results and relevant scientific literature [37,38,39].

The level of those molecules which are derived from systemic sources and are not affected by the preparation protocols, including MPO, ALCAM, CRP, Lipocalin-2, EMMPRIN, Osteopontin, IL-17A, CHI3L1, CD97, CD40L, was similar. From the elements of the complement system, C5a was present in the highest level in plasma, while C1qR1 was present in a similar concentration in all cases indicating that it was already activated after blood drawing regardless of the processing method. Platelet-derived pro-inflammatory cytokines and growth factors were all the highest in EDTA PRP compared to the other blood derivatives except for fibrinogen, which was high both in plasma and EDTA PRP compared to serum and HAS. This was due to the fibrin clot being separated from both HAS and serum after coagulation. Analyzing the concentration of anti-inflammatory cytokines in the samples, IL-1RA was elevated in EDTA PRP and HAS, and angiopoietin-1 was elevated in serum only. Interestingly the platelet-derived molecules were not increased in HAS or serum, although in both cases platelets are activated and release cytokines. This is probably due to the fact that serum is deprived of platelets and it contains only molecules which the platelets actively secrete. This is closer to the physiological conditions than disrupting all platelets and mixing their content into the plasma, as in the case of EDTA PRP activated by thrombin and calcium-chloride for example. The composition analysis of blood derivatives revealed that although the preparation method of EDTA PRP and HAS are similar, and they are typically considered interchangeable in clinical settings, there are marked differences. Most strikingly EDTA PRP contains excess EDTA, and as a consequence imbalanced ionic composition, higher growth factor content, and elevated pro-inflammatory cytokine content is present compared to HAS. According to previous results, EDTA PRP contains more angiogenic factors as well [31]. On the basis of our results so far, HAS eliminates the disadvantages of PRP preparation, such as the addition of EDTA, citrate, thrombin, or calcium chloride, which may cause side effects inside an injured tissue. HAS is free from activated platelets, which may be the reason why EDTA PRP is more pro-inflammatory than HAS. The presence of pro-inflammatory cytokines could further enhance inflammation in injured tissues. Further benefits of using HAS instead of EDTA PRP is the better cell proliferative effect on mesenchymal stem cells, osteoblasts, and osteoarthritic chondrocytes than EDTA PRP based on our previous results [32,34,35].

## 4. Materials and Methods

### 4.1. Isolation of Blood Derivates

**Figure 5:**
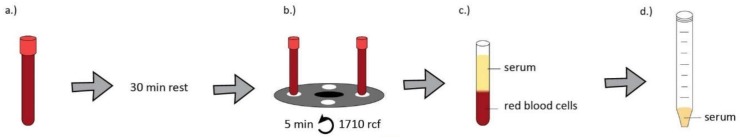
For serum isolation, whole blood was obtained from donors in VACUETTE^®^ 9 mL Z Serum C/A tubes (Greiner Bio-One, Kremsmünster, Austria). Blood was allowed to clot for 30 min (**a**) and centrifuged at 1710× *g* for 5 min at room temperature (**b**). The supernatant formed after centrifugation is called serum (**c**,**d**).

**Figure 6:**
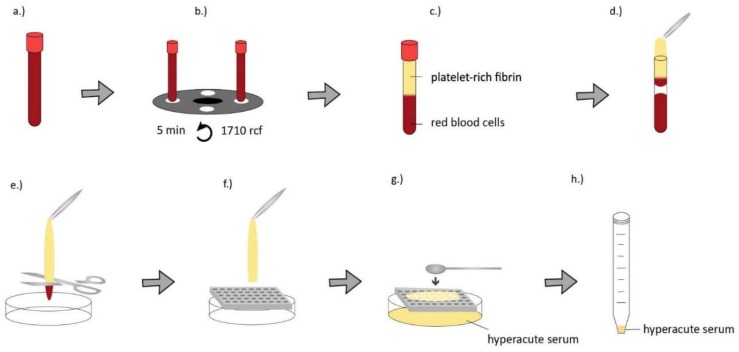
For hyperacute serum isolation, a whole blood sample was obtained from healthy donors (28–45 years) in VACUETTE^®^ 9 mL Z Serum C/A tubes (Greiner Bio-One) (**a**) and it was immediately centrifuged at 1710× *g* for 5 min at room temperature (**b**). After centrifugation two layers were formed in the tubes. The top layer was the platelet-rich fibrin clot and the bottom layer contains red blood cells (**c**). PRF (platelet-rich fibrin) as removed using sterile forceps in a biosafety cabinet (**d**), red blood cells at the bottom of the fibrin clot were cut away (**e**) and the clot was placed onto a 110 mm long, 75 mm wide custom-made plastic grid with 5 mm diameter holes on it. It was sterilized in an autoclave before use (**f**). The hyperacute serum was squeezed out from the PRF clot using a sterile spatula (**g**,**h**) [24].

**Figure 7:**
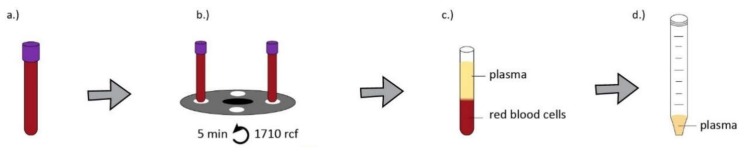
For PRP isolation, whole blood was obtained from donors in VACUETTE^®^ 9 mL K3 EDTA blood collection tubes (Greiner Bio-One) and VACUTTE 3.5 mL sodium citrate 3.2% blood collection tubes (Greiner Bio-One) (**a**) then centrifuged at 1710× *g* for 5 min at room temperature (**b**). The supernatant formed after centrifugation is called plasma (**c**,**d**).

**Figure 8:**
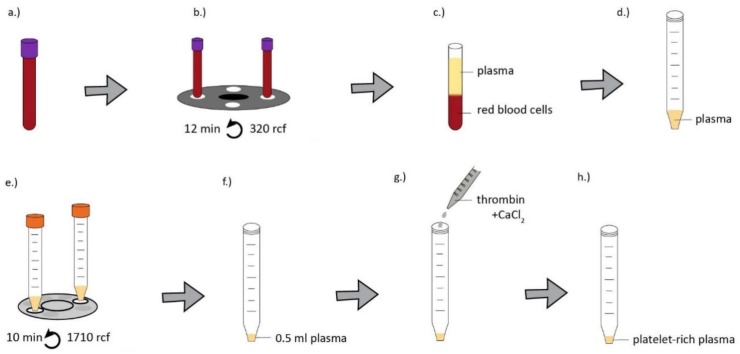
For PRP isolation, whole blood was obtained from donors in VACUETTE^®^ 9 mL K3 EDTA blood collection tubes (Greiner Bio-One) and VACUTTE 3.5 mL sodium citrate 3.2% blood collection tubes (Greiner Bio-One) (**a**) then centrifuged at 320 *g* for 12 min at room temperature (**b**). The platelet-rich layer above the buffy coat was aspirated and transferred into a 15 mL tube (**c**,**d**) and centrifuged at 1710× *g* for 10 minutes (**e**). The resulting platelet pellet was resuspended in the same volume as the isolated hyperacute serum from the same donor (**f**). PRP was activated by 10 IU thrombin and 10 mg calcium-chloride (Sigma-Aldrich, St. Louis, MO, USA) in case of both EDTA and citrate tubes (**g**,**h**).

Blood samples were obtained from healthy donors of both genders aged 24–45 years under IRB approval (IRB approval number 33106-1/2016/EKU, 12.07.2016.). The preparation method of serum, hyperacute serum, plasma and platelet-rich plasma is shown in Figure 5, Figure 6, Figure 7 and Figure 8.

### 4.2. Laboratory Testing of Blood Derivates

A Sysmex XN-1000 Sa-01 cell counter was used for the quantitative determination of red blood cells, leukocytes and platelet number in the serum and plasma fractions. The concentration of ions, that may interfere with EDTA and sodium citrate (calcium, magnesium, copper, zinc, iron, sodium, phosphate, and potassium) and the activity of ALP enzymes were measured using a Beckman Coulter AU5800 automated laboratory machine (*n* = 4).

### 4.3. Comprehensive Protein Analysis

In order to quantify the cytokine and angiogenesis-related protein content of EDTA PRP and plasma serum a HAS Proteome Profiler Human Angiogenesis Array Kit (55 protein, R&D Systems, #ARY007) (R&D Systems Inc., Minneapolis, MN, USA) [35] and Proteome Profiler Human XL Cytokine Array Kit (102 protein R&D Systems, #ARY022) were used according to the manufacturer’s instructions. Some cytokines were included in both the Proteome Profiler Human Angiogenesis Array Kit and Proteome Profiler Human XL Cytokine Array Kit, in these cases we used the mean of the results. In total, 138 different cytokines were measured by the two different kits. Proteome Profiler individual protein levels were measured as spot intensities on the blots using Fiji Image J 1.47 (South Bend, IN, USA) and Adobe Photoshop CC 2015.5 software (San Jose, CA, USA). The results were expressed in % compared to the arbitrary unit of the highest AU value, which belonged to ANG. Thus, the combined protein level of ANG among the samples was considered to be 100% (*n* = 8).

### 4.4. Quantitative Protein Measurement by Multiplex Immunoassay and ELISA Assays

The key proteins, where clear differences were found during the proteome profiler measurements, were quantified using custom Human Magnetic Luminex Assay (CHI3L1, C5a, EGF, CD40L, VEGF, CRP, Il-17a, Osteopontin, Angiopoetin, IL-1RA, EMMPRIN, Lipocalin-2, Pentraxin-3, PDGF-AA, PDGF-BB) (R&D Systems Inc.) or ELISA assays (MPO, ALCAM, CD97, C1qR1, TGF-beta, Fibrinogen, Thrombospondin-1, CXCL-5) (Abcam, Cambridge, UK) (*n* = 8)

### 4.5. Statistical Analysis

One-way analysis of variance (ANOVA) was performed with a Tukey post hoc test to compare differences between the groups. The significance level was *p* > 0.05, where * means that *p* is between 0.01 and 0.05, ** means that *p* is between 0.01 and 0.001, and *** means that *p* is lower than 0.001. Prism 7 software (Irvine, CA, USA) was used for statistical analysis. Data are presented as mean ± SEM.

## 5. Conclusions

General trends can be observed in the composition changes of the blood products that were investigated. These trends are based on the characteristics of the additives and the steps of the production methods. For the preparation of PRPs calcium-chloride, thrombin, citrate or EDTA is added, which has a negative effect on the composition of these blood products. The exact mechanism of HAS on cells is not regenerative, only acts as a supplement but with the use of HAS the negative effects originating from the PRP’s preparation methods can be circumvented, while maintaining the beneficial influence of PRP on cells and tissues in vitro. Our further aims include comparing the effect of HAS, EDTA and citrate PRP in clinical studies, whicht can be differ from the in vitro results.

## Abbreviations

ALCAMactivated leukocyte cell adhesion moleculeALPalkaline phosphataseCD40Lcluster of differentiation 40 ligandCD97cluster of differentiation 97CHI3L1chitinase 3-like protein 1CRPC-reactive proteinCXCL-5chemokine (C-X-C motif) ligand 5C1qR1complement component 1 Q subcomponent receptor 1C5acomplement component 5aEDTAethylenediaminetetraacetic acidEGFepidermal growth factorEMMPRINextracellular matrix metalloproteinase inducerELISAenzyme-linked immunosorbent assayHAShyperacute serumIL-17interleukin-17IL-1RAinterleukin-1 receptor antagonistMSCmesenchymal stem cellTGF-betatransforming growth factor betaPDGF-AAplatelet-derived growth factor AAPDGF-BBplatelet-derived growth factor BBPRFplatelet-rich fibrinPRPplatelet-rich plasmaSPRFserum from platelet-rich fibrinVEGFvascular endothelial growth factor

## Supplementary Materials

Supplementary materials can be found at http://www.mdpi.com/1422-0067/20/3/721/s1.
