# Supplementary material for: The Composition of Hyperacute Serum and Platelet-Rich Plasma Is Markedly Different despite the Similar Production Method
Source: Int J Mol Sci. 2019 Feb 8;20(3):721. doi: 10.3390/ijms20030721 (PMC6386965; doi:10.3390/ijms20030721)
Supplement: Supplementary file 1 [file ijms-20-00721-s001.pdf]

Supplementary materials

Table S1.

Abbreviation of the proteins	Name of the proteins	Protein level (AU) %			
		PRP	HAS	Serum	Plasma
**Activin A**	Activin A	1.63	1.37	2.17	2.60
**ADAMTS-1**	A disintegrin and metalloproteinase with thrombospondin motifs 1	0.10	0.00	0.00	0.00
**Adiponectin**	Adiponectin	13.16	15.41	8.07	8.49
**ACN**	Aggrecan	11.48	10.38	4.93	5.49
**ANG**	Angiogenin	37.28	22.26	19.65	20.73
**Ang-1**	Angiogenin-1	4.95	2.39	14.26	0.30
**Ang-2**	Angiogenin-2	0.30	0.03	0.00	0.05
**ANGPT1**	Angiopoietin-1	5.05	2.98	0.95	0.31
**ANGPT2**	Angiopoietin-2	0.00	0.37	0.04	0.14
**Angiostatin/Plasminogen**	Angiostatin/Plasminogen	0.64	0.29	0.32	0.72
**AR**	Androgen receptor	0.00	0.00	0.00	0.02
**Artemin**	Artemin	0.03	0.00	0.01	0.00
**Average Reference Spots**	Average Reference Spots	16.08	16.08	16.08	16.08
**BAFF**	B-cell activating factor	6.95	6.70	1.67	1.61
**BDNF**	Brain-derived neurotrophic factor	15.89	0.62	1.40	0.24
**C5/C5a**	Complement component 5/Complement component 5a	8.01	5.88	0.74	0.35
**CD14**	Cluster of differentiation 14	7.88	3.85	0.56	0.43
**CD30**	Cluster of differentiation 30	0.00	0.28	0.18	0.13
**CD40 Ligand/TNFSF5**	Cluster of differentiation 4 Ligand	5.40	1.26	0.28	0.17
**CFD**	Complement factor D	11.62	13.33	6.49	6.52
**CHI3L1**	Chitinase 3-like protein 1	15.52	17.37	13.43	13.81
**Cripto-1**	Epidermal growth factor-Cripto-FRL1-Cryptic	0.00	0.35	0.15	0.21
**CRP**	C-reactive protein	14.42	18.43	11.92	14.17
**CXCL16**	Chemokine (C-X-C motif) ligand 16	19.84	13.25	11.86	13.03
**Cystatin C**	Cystatin C	9.96	8.58	2.93	2.30
**Dkk-1**	Dickkopf-related protein 1	11.76	0.97	0.24	0.11
**DPPIV**	Dipeptidyl peptidase IV.	14.90	9.58	11.14	11.88
**EGF**	Epidermal growth factor	12.83	0.37	0.08	0.17
**EG-VEGF**	Endocrine-gland-derived vascular endothelial growth factor	0.00	0.06	0.01	0.01
**EMMPRIN**	Expression of extracellular matrix metalloproteinase inducer	13.56	8.74	3.05	3.82
**ENA-78**	Epithelial cell-derived neutrophil-activating peptide 78	12.36	7.98	0.94	0.88
**ENG**	Endoglin	10.99	11.43	4.38	4.32
**Endostatin/Collagen XVIII**	Endostatin/Collagen XVIII	0.75	4.86	6.54	10.11
**ET-1**	Endothelin-1	0.00	0.05	0.00	0.09
**FASL**	Fas Ligand	0.00	0.70	0.15	0.40
**FGF basic**	Fibroblast growth factor basic	0.00	1.96	0.32	0.59

**FGF-1**	Fibroblast growth factor 1	0.13	0.02	0.00	0.08
**FGF-19**	Fibroblast growth factor 19	3.49	1.00	0.23	0.11
**FGF-2**	Fibroblast growth factor 2	0.00	0.00	0.00	0.00
**FGF-4**	Fibroblast growth factor 4	0.00	0.02	0.00	0.00
**FGF-7**	Fibroblast growth factor 7	0.07	0.01	0.00	0.03
**FLT3L**	FMS-like tyrosine kinase 3 ligand	0.00	0.34	0.02	0.00
**G-CSF**	Granulocyte colony-stimulating factor	0.00	0.40	0.00	0.05
**GDF-15**	Growth differentiation factor 15	3.87	1.89	0.01	0.37
**GDNF**	Glial cell line-derived neurotrophic factor	0.00	0.00	0.00	0.00
**GH**	Growth hormone	0.00	0.04	0.01	0.04
**GM-CSF**	Granulocyte-macrophage colony-stimulating factor	0.00	0.04	0.00	0.01
**CXCL-1/GRO-a**	Hemokine (C-X-C motif) ligand/Growth-regulated alpha protein	3.40	0.08	0.02	0.00
**HB-EGF**	Heparin binding EGF-like growth factor	6.58	0.06	0.17	0.00
**HGF**	Hepatocyte growth factor	0.05	0.00	0.00	0.00
**ICAM-1**	Intercellular Adhesion Molecule 1	6.57	4.22	0.39	0.53
**IFNγ**	Interferon gamma	0.00	0.16	0.03	0.03
**IGFBP-1**	Insulin-like growth factor-binding protein 1	37.07	14.55	13.95	14.53
**IGFBP-2**	Insulin-like growth factor-binding protein 2	18.65	9.23	10.56	13.11
**IGFBP-3**	Insulin-like growth factor-binding protein 2	25.27	16.14	14.33	16.55
**IL-10**	Interleukine 10	0.00	0.00	0.02	0.03
**IL-11**	Interleukine 11	0.00	0.61	0.00	0.06
**IL-12 p70**	Interleukine 12	0.00	0.42	0.03	0.02
**IL-13**	Interleukine 13	0.00	0.09	0.03	0.02
**IL-15**	Interleukine 15	0.00	0.05	0.00	0.00
**IL-16**	Interleukine 16	4.12	0.06	0.09	0.01
**IL-17a**	Interleukine 17	2.79	1.07	0.06	0.21
**IL-18 Bpa**	Interleukine 18	1.61	1.18	0.02	0.04
**IL-19**	Interleukine 19	2.57	0.00	0.03	0.04
**IL-1α**	Interleukine 1 alpha	0.00	0.22	0.00	0.04
**IL-1β**	Interleukine 1 beta	0.00	0.00	0.00	0.00
**IL-1RA**	Interleukine 1 receptor antagonist	9.47	0.25	0.01	0.00
**IL-2**	Interleukine 2	0.00	0.09	0.00	0.00
**IL-22**	Interleukine 22	0.00	0.07	0.02	0.01
**IL-23**	Interleukine 23	0.00	0.19	0.00	0.05
**IL-24**	Interleukine 24	0.00	0.10	0.10	0.00
**IL-3**	Interleukine 3	1.12	0.00	0.00	0.00
**IL-31**	Interleukine 31	0.00	0.00	0.01	0.07
**IL-32α/β/γ**	Interleukine 32	0.00	0.05	0.09	0.00
**IL-33**	Interleukine 33	0.00	0.01	0.02	0.05
**IL-34**	Interleukine 34	0.00	0.15	0.02	0.03
**IL-37**	Interleukine 37	0.00	0.14	0.00	0.07
**IL-4**	Interleukine 4	0.00	0.00	0.01	0.00
**IL-5**	Interleukine 5	0.00	0.16	0.02	0.00
**IL-6**	Interleukine 6	0.00	0.04	0.00	0.13
**IL-8**	Interleukine 8	0.07	0.00	0.00	0.04

**IP-40**	Interferon gamma inducible protein 40	6.21	1.09	0.09	0.08
**I-TAC/CXCL-11**	Interferon-inducible T-cell alpha chemoattractant/ Chemokine (C-X-C motif) ligand 11	0.00	0.10	0.00	0.02
**KLK3**	kallikrein related peptidase 3	4.27	0.81	0.09	0.00
**LAP(TGF-β1)**	Latency-associated peptide (Transforming rowth factor beta 1)	25.20	0.00	0.02	0.00
**Leptin**	Leptin	13.13	12.66	13.53	13.74
**LIF**	Leukemia inhibitory factor	0.00	0.04	0.00	0.00
**LCN2**	Lipocalin-2	12.07	13.31	8.43	5.35
**MCP-1/CCL-2**	Monocyte chemoattractant protein-1/C-C motif chemokine ligand 2	0.00	0.00	0.00	0.01
**MCP-3/CCL-7**	Monocyte chemoattractant protein-3/C-C motif chemokine ligand 7	0.00	0.07	0.05	0.00
**M-CSF**	Macrophage colony-stimulating factor	0.00	0.01	0.02	0.00
**MIF**	Macrophage migration inhibitory factor	8.23	4.17	0.74	0.68
**CXCL-9/MIG**	Monokine induced by gamma/ Chemokine (C-X-C motif) ligand 9	0.00	0.10	0.01	0.00
**CCL-3/MIP-1α**	Chemokine (C-C motif) ligand 3/ macrophage inflammatory protein 1-alpha	0.07	0.01	0.04	0.07
**MIP-1α (CCL-3)/MIP-1β (CCL-4)**	Chemokine (C-C motif) ligand 3/ macrophage inflammatory protein 1-alpha/Chemokine (C-C motif) ligand 4/ macrophage inflammatory protein 1-beta	0.00	0.00	0.25	0.07
**MIP-3α/CCL-20**	Chemokine (C-C motif) ligand 20/ macrophage inflammatory protein 3-alpha	0.00	0.00	0.00	0.00
**MIP-3β/CCL-19**	Chemokine (C-C motif) ligand 19/ macrophage inflammatory protein 3-alpha	0.00	0.50	0.06	0.03
**MMP-8**	Matrix metallopeptidase 8	1.96	1.50	6.06	1.39
**MMP-9**	Matrix metallopeptidase 9	23.92	15.71	19.34	17.07
**MPO**	Myeloperoxidase	0.00	0.34	0.03	0.06
**NRG1-β1**	Human Neuregulin-1 beta 1	0.00	0.00	0.04	0.00
**OPN**	Osteopontin	6.40	1.60	0.42	0.83
**PD-ECGF**	Platelet-derived endothelial cell growth factor	0.04	0.07	0.04	0.02
**PDGF-AA**	Platelet-derived growth factor AA	16.10	13.61	15.74	6.53
**PDGF-AA/AB**	Platelet-derived growth factor AA/AB	16.47	12.88	12.34	2.52
**Persephin**	Persephin	0.04	0.05	0.00	0.00
**PF4**	Platelet factor 4	0.00	3.59	2.06	13.71
**PIGF**	Phosphatidylinositol-glycan biosynthesis class F protein	0.09	0.02	0.00	0.00
**Prolactin**	Prolactin	16.32	9.95	12.57	11.57
**PTX 3**	Pentraxin-3	6.97	1.77	4.28	3.72
**RAGE**	Receptor for Advanced Glycation Endproducts	0.00	0.46	0.09	0.26
**RANTES/CCL-5**	Regulated on activation. normal T cell expressed and secreted/Chemokine (C-C motif) ligand 5	10.07	11.29	6.50	5.43
**RBP4**	Retinol binding protein 4	18.21	18.20	20.90	17.71
**RLN2**	Relaxin-2	0.00	0.16	0.02	0.08
**Resistin**	Resistin	17.56	9.42	2.50	1.68
**SDF-1a**	Stromal cell-derived factor-1	5.74	1.42	0.25	0.15
**Serpin B5**	Serpin family B member 5	0.00	0.00	0.00	0.00
**Serpin E1**	Serpin family E member 1	5.14	21.37	18.32	18.01

**Serpin F1**	Serpin family F member 1	0.05	0.00	0.01	0.04
**SHBG**	Sex hormone binding globulin	8.31	5.60	1.80	1.86
**ST2**	Suppression of tumorigenicity 2	6.15	4.99	1.22	1.65
**TARC/CCL-17**	Thymus and activation regulated chemokine/Chemokine (C-C motif) ligand 17	10.67	0.81	0.24	0.18
**TF**	Transferrin	0.00	0.00	0.00	0.09
**TFF3**	Trefoil factor 3	8.76	5.31	1.18	1.83
**TfR**	Transferrin Receptor	0.00	0.69	0.28	0.30
**TGF-α**	Transforming growth factor alpha	0.00	0.06	0.07	0.12
**THBS1**	Thrombospondin-1	0.00	3.49	0.92	9.63
**THBS2**	Thrombospondin-2	0.10	0.14	0.11	0.00
**TIMP-1**	Tissue inhibitor of metalloproteinase 1	23.13	24.26	21.31	20.46
**TIMP-4**	Tissue inhibitor of metalloproteinase 4	35.95	21.25	20.46	18.59
**TNF-α**	Tumor necrosis factor alpha	0.00	0.45	1.32	1.41
**Upa**	Urokinase-type plasminogen activator	3.83	3.48	5.37	5.33
**Upar**	Urokinase-type plasminogen activator receptor	6.41	0.79	0.10	0.06
**VASH1**	Vasohibin	0.13	0.02	0.00	0.00
**VEGF**	Vascular Endothelial Growth Factor	11.49	1.51	5.25	1.93
**VEGF-C**	Vascular Endothelial Growth Factor C	0.03	0.00	0.00	0.00
**Vitamin D BP**	Vitamin D binding protein	2.87	13.27	0.00	0.13
